# Effects of dual bronchodilation on right ventricular function and troponin-I in newly diagnosed, moderate-to-severe chronic obstructive pulmonary disease: a prospective real-world observational study

**DOI:** 10.1177/17534666261452491

**Published:** 2026-06-24

**Authors:** Ieva Dimiene, Gintare Neverauskaite-Piliponiene, Paulius Bucius, Paulius Simkus, Lina Padervinskiene, Airidas Rimkunas, Egle Ereminiene, Skaidrius Miliauskas

**Affiliations:** Department of Pulmonology, Medical Academy, Lithuanian University of Health Sciences, A. Mickeviciaus Street 9, Kaunas LT-44307, Lithuania; Heart Centre, Hospital of Lithuanian University of Health Sciences Kauno Klinikos, Kaunas, Lithuania; Heart Centre, Medical Academy, Lithuanian University of Health Sciences, Kaunas, Lithuania; Department of Radiology, Hospital of Lithuanian University of Health Sciences Kauno Klinikos, Kaunas, Lithuania; Department of Radiology, Medical Academy, Lithuanian University of Health Sciences, Kaunas, Lithuania; Laboratory of Pulmonology, Department of Pulmonology, Medical Academy, Lithuanian University of Health Sciences, Kaunas, Lithuania; Heart Centre, Medical Academy, Lithuanian University of Health Sciences, Kaunas, Lithuania; Institute of Cardiology, Lithuanian University of Health Sciences, Kaunas, Lithuania; Department of Pulmonology, Medical Academy, Lithuanian University of Health Sciences, Kaunas, Lithuania

**Keywords:** COPD, RV, strain, tiotropium/olodaterol, troponin-I

## Abstract

**Background:**

Chronic obstructive pulmonary disease (COPD) is associated with right ventricular (RV) dysfunction and subclinical myocardial injury. The effects of dual bronchodilation on RV function and cardiac injury biomarkers remain limited.

**Objectives:**

To evaluate 12-week changes in pulmonary function, RV function, and serum troponin-I in newly-diagnosed COPD patients receiving tiotropium/olodaterol as routine clinical care and to assess differences in RV function and troponin-I between COPD and non-COPD controls with comparable cardiovascular diseases (CVDs).

**Design:**

Prospective real-world observational study.

**Methods:**

Treatment-naïve patients with moderate-to-severe COPD were assessed at baseline and after 12 weeks of treatment. Evaluations included spirometry, plethysmography, two-dimensional and speckle-tracking transthoracic echocardiography, cardiac magnetic resonance imaging (MRI), and troponin-I. Baseline RV measurements and troponin-I were compared between COPD (*n* = 47) and controls (*n* = 23).

**Results:**

COPD patients had lower RV fractional area change and RV free-wall strain (FWS) than controls (*p* < 0.001 and *p* = 0.018, respectively). Troponin-I was above the limit of detection in 93.6% of COPD and in 56.5% of controls (*p* < 0.001). COPD treatment increased forced expiratory volume in 1 second (FEV_1_) and forced vital capacity while reducing residual volume-to-total lung capacity (TLC) and functional residual capacity (FRC)-to-TLC (*p* < 0.05). RV global longitudinal strain (GLS) improved from 21.7 ± 2.6% to 23.0 ± 2.8%, and RV-FWS from 24.2 ± 3.3% to 26.1 ± 3.6% (both *p* < 0.05) in echocardiography (*n* = 29). ΔRV-GLS correlated positively with ΔFEV_1_ and inversely with ΔFRC/TLC (*p* < 0.05). In the moderate COPD subgroup, RV-GLS in MRI changed from 22.6 ± 6.1% to 25.5 ± 7.5% (*p* = 0.037), and troponin-I decreased from 1.23 pg/mL (IQR 1.03–1.56) to 1.09 pg/mL (IQR 0.95–1.20; *p* = 0.049).

**Conclusion:**

COPD affects RV function and troponin-I regardless of concomitant CVDs. Initiation of dual bronchodilation is associated with beneficial effects on RV strain and troponin-I in patients with COPD. Because of the small sample size, these findings should be interpreted cautiously and confirmed in larger studies.

**Trial registration:**

ClinicalTrials.gov; ID NCT06072690; https://clinicaltrials.gov/study/NCT06072690.

## Introduction

Chronic obstructive pulmonary disease (COPD) is a respiratory condition associated with significant systemic manifestations, among which cardiovascular involvement is one of the most important.^1,2^ COPD and cardiovascular diseases (CVDs) share risk factors and have common pathobiological pathways. These shared mechanisms include systemic inflammation, oxidative stress, and endothelial dysfunction, with additional contributions from metabolic dysregulation, all of which may further increase cardiovascular risk in COPD.^3,4^ Recent evidence also suggests that smoking is independently associated with coronary microvascular dysfunction, supporting microvascular injury as a potential link between pulmonary pathologies and CVDs.^5^ In addition, abnormal respiratory physiology contributes to the development of heart failure.^6^

There are data indicating that COPD treatment regimens consisting of both long–acting β_2_–agonist (LABA) and long-acting muscarinic antagonist (LAMA), also including triple therapy, are associated with a higher risk of cardiovascular events, while other studies have demonstrated that these treatment options do not confer an increased risk compared with other regimens.^7–9^ Moreover, studies have demonstrated that COPD treatment with dual bronchodilation positively affects cardiac volumes, as assessed by cardiac magnetic resonance imaging (MRI) or two-dimensional (2D) transthoracic echocardiography (TTE).^10–12^

Recently, cardiac strain has emerged as a sensitive biomarker for early detection of cardiac dysfunction across various conditions, including COPD.^13,14^ In patients with COPD, changes in intrathoracic pressure increase resistance in the pulmonary circulation. The resulting rise in afterload leads to concentric hypertrophy of the right ventricle (RV), which gradually impairs cardiac pump function, resulting in the development of right heart failure. Subsequently, concentric hypertrophy affects diastolic relaxation and may impair systolic strain, while conventional indices of RV function might remain unchanged.^14,15^ Evaluation of RV structure and function can be performed using both—2D TTE and cardiac MRI.^16^ In everyday clinical practice, 2D TTE is the first-line imaging modality; however, its performance in patients with COPD might be challenging because of lung hyperinflation and air trapping, resulting in suboptimal image quality in approximately 10%–50% of cases, depending on the severity of airflow limitation.^16,17^ Although cardiac MRI is considered the most accurate modality for RV evaluation, its use is limited by restricted availability, technical complexity, high cost, and contraindications, including claustrophobia.^18–21^ Several studies have found that patients with COPD present less negative (worse) ventricular strain when compared with healthy controls.^15,22,23^ Nevertheless, to date, no published studies have specifically evaluated the effects of particular COPD treatment on ventricular strain.

Another cardiovascular biomarker of interest in COPD is serum troponin-I. Data from the large cohort showed that RV dysfunction was an independent determinant of higher high-sensitivity troponin-I concentrations (⩾6 ng/L) in patients with stable COPD, although only 1.8% had levels above the 99th-percentile reference limit (27 ng/L).^24^ This supports the concept that RV dysfunction may contribute to chronic low-grade myocardial damage, thereby influencing serum troponin-I concentrations.

This prospective, observational real-world study had two primary endpoints: (a) to evaluate changes in RV global longitudinal strain (GLS) and RV free-wall strain (FWS) assessed by speckle-tracking 2D TTE and cardiac MRI; and (b) to assess changes in serum troponin-I after 12 weeks of treatment with tiotropium/olodaterol (TIO/OLO) 5/5 µg fixed-dose combination administered once daily as part of routine clinical care in patients with newly diagnosed, moderate-to-severe COPD. Secondary endpoints included changes in conventional 2D TTE parameters—RV diameter, RV fractional area change (FAC), and tissue Doppler–derived tricuspid annular systolic velocity (S′)—during treatment, as well as comparisons of conventional 2D TTE measurements of RV function and serum troponin-I levels between COPD and non-COPD individuals.

## Methods

### Study design and participants

**Figure 1.**
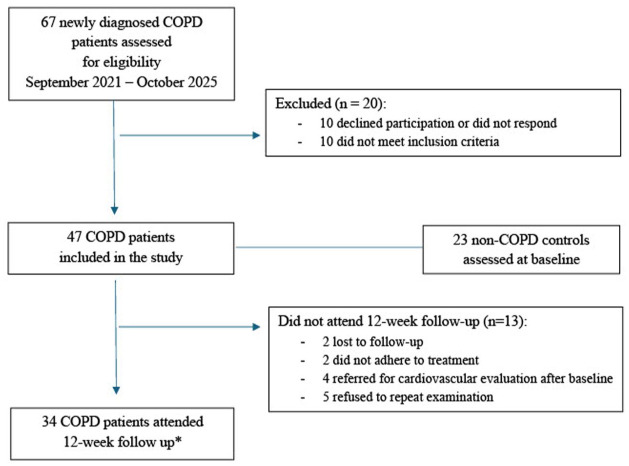
Flow diagram of participant selection and follow-up. *Detailed information on incomplete examinations is described in the Methods section.

We performed a prospective, real-world observational study with a duration of 12 weeks at the Hospital of Lithuanian University of Health Sciences Kauno Klinikos. Newly diagnosed COPD patients were identified and assessed for eligibility as they presented at the Department of Pulmonology during routine clinical practice between September 2021 and October 2025. No formal sample size or study power calculation was performed prior to the study; all eligible treatment-naïve COPD patients who agreed to participate were included. A total of 47 newly diagnosed treatment-naïve patients with moderate-to-severe COPD (study group) and 23 participants of comparable age, sex, body mass index (BMI), and CVDs profile without respiratory diseases (control group) were enrolled. Participant selection and follow-up are presented in a flow diagram (Figure 1). The inclusion and exclusion criteria for COPD patients have been described previously.^25^ Control group included patients aged ⩾40 years without respiratory diseases. Stable cardiovascular diseases (except heart failure with reduced ejection fraction) were not considered exclusion criteria in either group.

The study group consisted of COPD patients to whom TIO/OLO 5/5 µg was prescribed as part of routine clinical care. The diagnosis of COPD was confirmed by a pulmonologist at our center, according to the Global Initiative for Chronic Obstructive Lung Disease (GOLD) criteria, based on post-bronchodilator spirometry demonstrating a forced expiratory volume in 1 second (FEV_1_)/forced vital capacity (FVC) ratio <0.7.^26^ No exacerbations were documented prior to study inclusion, and patients were clinically stable at the time of enrollment. No additional pharmacological treatments were initiated during the study period for either COPD or concomitant comorbidities.

COPD patients underwent spirometry, body plethysmography, 2D and speckle-tracking TTE, cardiac MRI, and serum troponin-I testing at the time of diagnosis and at 12 weeks, using standardized measurement protocols and consistent assessment methods. The same protocols and assessment methods were applied in the control group. Follow-up assessments were conducted 2 h after the morning dose. Changes in these measurements were evaluated over the 12-week study period in the combined group of moderate and severe COPD patients, as well as separately in patients with moderate COPD. The total of 34 subjects in the COPD group attended the 12-week follow-up visit. The remaining patients either did not adhere to treatment recommendations, required referral for cardiological evaluation and treatment, refused to repeat the examinations, or were lost to follow-up. Baseline characteristics were generally similar between completers and non-completers (Supplemental Table 1). Not all cardiovascular measurements were available for all 34 patients who completed the study. Detailed descriptions of the methodologies for each examination and analysis are provided in separate subsections.

The control group underwent one-time testing consisting of spirometry, body plethysmography, 2D and speckle-tracking TTE, and serum troponin-I concentration. Baseline findings were then compared with those of the study group.

### Pulmonary function testing

We measured FEV_1_, FVC, and the FEV_1_-to-FVC ratio with a Ganshorn spirometry device (Ganshorn Medizin Electronic, Niederlauer, Germany). Body plethysmography was conducted in a Ganshorn Power Cube Body+ plethysmography chamber (Ganshorn Medizin Electronic, Niederlauer, Germany). The following parameters of body plethysmography were assessed: residual volume, total lung capacity (TLC), residual volume-to-TLC, functional residual capacity (FRC), and FRC/TLC. For each parameter, results were expressed as absolute values (L or %) and as a percentage of predicted values (% pred.).

### Two-dimensional and speckle-tracking transthoracic echocardiography

We performed a 2D and speckle-tracking TTE using an ultrasound system (model EPIQ7, Philips Medical Systems). Echocardiography was performed before initiating COPD treatment as a baseline and repeated during the 12-week follow-up visit by an experienced cardiologist. The protocol involved high-quality apical and parasternal views. Measurements were obtained according to the latest guidelines for echocardiographic assessment of the right heart.^27^

RV diameter measurements were obtained from an RV-focused apical 4-chamber view at end-diastole, measured at the basal level, and aligned longitudinally with the tricuspid annular plane. RV area parameters were obtained from the RV-focused 4-chamber apical view. By tracing the endocardial border from the lateral tricuspid annulus along the right ventricular wall to the apex and along the interventricular septum to the medial tricuspid annulus. Measurements were made at end-diastole to obtain RV end-diastolic area (RVEDA), as well as at end-systole to obtain RV end-systolic area (RVESA). RV-FAC was used to assess the global systolic function of the RV. RV-FAC was calculated by dividing the RVEDA and RVESA difference by RVEDA. RV-S′ was obtained in RV-focused 4-chamber view, placing the sample at the base of the RV free wall. Tricuspid annular plane systolic excursion (TAPSE) was excluded from measurements due to a lack of optimal acoustic window; we chose to measure only RV-S′.

RV-GLS and RV-FWS were assessed using 2D speckle-tracking TTE (Supplemental Figure 1). Although RV strain values are conventionally expressed as negative values, with more negative values indicating better results, absolute values were used in the present study to facilitate statistical analysis, such that higher values reflect better RV function.

Due to suboptimal acoustic windows related to lung hyperinflation, not all measurements could be obtained with adequate image quality. At baseline, FAC was successfully measured in 44 patients, while RV-GLS and RV-FWS were obtained in 41 patients. At the 12-week follow-up, FAC measurements were available in 28 of the 34 patients, and RV strain parameters were obtained in 29 patients. In the control group, all 23 patients had high-quality echocardiographic images, and all measurements were obtained.

### Cardiac magnetic resonance imaging with feature tracking

All patients were scanned with the same MRI scanner (3.0 T Siemens Skyra, Siemens Medical Solutions; Erlangen, Germany). Cardiac MR images were analyzed by an experienced radiologist using a commercial feature tracking software package (Medis Suite, version 4.0.62.4, Medical Imaging System, Leiden, The Netherlands). All RV parameters were measured from balanced steady-state free precession cine images.

Endocardial RV borders were semi-automatically traced at end-diastole and end-systole in the short-axis stack. From these contours, the software calculated all volumetric and functional parameters: end-diastolic volume (EDV), end-systolic volume (ESV), stroke volume (SV), and ejection fraction (EF). Volumes were indexed (i) for body surface area (BSA). RV-GLS and RV-FWS were obtained using 4-chamber views (Supplemental Figure 2). Endocardial contours of the RV were marked manually in the end-diastole. Contours were drawn automatically in the end-systolic phase and adjusted manually if needed. While traditionally strains are expressed in negative values (the more negative value indicating the better result), in our study, strains were expressed in absolute values to ease the statistical analysis.

Changes in cardiac MRI parameters from baseline to 12-week follow-up were evaluated in 29 out of the 34 patients who completed the study. Four patients could not undergo cardiac MRI because of contraindications, including claustrophobia; one patient had low-quality images due to artifacts.

### Serum troponin-I concentration analysis

Serum troponin-I concentration was quantified using an enzyme-linked immunosorbent assay (ELISA) with the Human Cardiac Troponin-I (TNNI3) ELISA Kit (Invitrogen™, Thermo Fisher Scientific, Waltham, MA, USA), according to the manufacturer’s instructions. This assay was performed in the Laboratory of Pulmonology, the Department of Pulmonology, Lithuanian University of Health Sciences. Frozen serum samples were used for analysis. The lower limit of detection (LOD) for human troponin-I in this assay is 0.38 pg/mL. Values below the LOD were imputed as half the LOD (0.19 pg/mL).

Serum troponin-I concentration was measured in all 47 patients with COPD at baseline and in 32 patients at the 12-week follow-up. Follow-up samples from two patients were not evaluable because of hemolysis. In the control group, serum troponin-I was measured once in all 23 patients. Serum troponin-I was categorized as below or above the LOD for between-group comparisons.

### Statistical analysis

We used IBM SPSS Statistics for Windows, version 30.0.0.0 (IBM Corp., Armonk, NY, USA) for statistical analysis. Comparisons between study subjects and the control group were performed using the independent *t*-test for normally distributed variables and the Mann–Whitney for non-normally distributed variables. The chi-square test was used for categorical variables. Changes in variables from baseline to 12 weeks of treatment in COPD patients were calculated using paired samples *t*-test for normally distributed variables and the Wilcoxon signed-rank test for non-normally distributed variables. Quantitative variables are presented as mean ± standard deviation (SD) or median (interquartile range (IQR)), as appropriate. When evaluating correlations between the changes (deltas, Δ) of cardiovascular biomarkers and pulmonary function tests, Pearson’s correlation was used for normally distributed data and Spearman’s correlation—for non-normally distributed data. Analyses were performed using available data for each variable with no imputation of missing values. No adjustment for confounding was performed. Results were statistically significant when *p* < 0.05.

The reporting of this study conforms to the Strengthening the Reporting of Observational Studies in Epidemiology (STROBE) statement (Supplemental Table 2).^28^

## Results

### Characteristics of study and control groups

The study group included 47 COPD patients, and the control group consisted of 23 non-COPD patients. Both groups were of comparable age, sex, BMI, and CVD profile. The main characteristics of study and control subjects are presented in Table 1.

**Table 1.** Characteristics of study and control group subjects.

Characteristic	COPD (*n* = 47)	Non-COPD (*n* = 23)	*p*-Value*
**Demographics**			
Age, years	61.1 ± 8.0	58.8 ± 8.1	0.253
Sex (male : female)	38:9	15:8	0.152
BMI, kg/m^2^	27.3 ± 4.8	29.1 ± 5.2	0.155
Smoking history, pack-years	30 (15–47)	0 (0–16)	<0.001*
**Pulmonary function**			
FEV_1_ (L)	1.96 ± 0.58	3.43 ± 0.77	<0.001*
FEV_1_ (% pred.)	58.79 ± 13.58	102.87 ± 12.37	<0.001*
FVC (L)	3.37 ± 0.87	4.31 ± 0.92	<0.001*
FVC (% pred.)	78.19 ± 14.03	100.52 ± 11.16	<0.001*
Residual volume (L)	3.61 ± 0.93	2.51 ± 0.52	<0.001*
Residual volume (% pred.)	153 (131–174)	108 (97–123)	<0.001*
Residual volume-to-TLC (%)	51.64 ± 7.55	37.32 ± 6.17	<0.001*
Residual volume-to-TLC (% pred.)	133 (124–147)	99 (91–107)	<0.001*
FRC (L)	4.30 ± 1.11	3.30 ± 0.79	<0.001*
FRC (% pred.)	125.81 ± 30.98	98.61 ± 20.42	<0.001*
FRC/TLC (%)	61.90 ± 8.66	49.20 ± 7.72	<0.001*
FRC/TLC (% pred.)	119.91 ± 15.97	96.61 ± 15.80	<0.001*
TLC (L)	6.95 ± 1.40	6.81 ± 1.23	0.684
TLC (% pred.)	104.34 ± 17.29	104.39 ± 12.70	0.990
**GOLD stage**			
GOLD 2 (%)	41 (87.2)	N/A	N/A
GOLD 3 (%)	6 (12.8)	N/A	N/A
**Blood eosinophil count**			
<300 cells/µL	31 (66.0)	N/A	N/A
⩾300 cells/µL	16 (34.0)	N/A	N/A
**Cardiovascular diseases**			
Arterial hypertension, *n* (%)	31 (66.0)	14 (60.9)	0.676
Stable ischemic cardiac disease, *n* (%)	6 (12.8)	4 (17.4)	0.719
Dyslipidemia, *n* (%)	22 (46.8)	12 (52.2)	0.673
Arrhythmias in the past, *n* (%)	4 (8.5)	2 (8.7)	1

* *p*-Value < 0.05 indicates statistically significant results.

BMI, body mass index; FEV_1_, forced expiratory volume in 1 second; FRC, functional residual capacity; FVC, forced vital capacity; GOLD, Global Initiative for Chronic Obstructive Lung Disease; N/A, not applicable; % pred., percentage of predicted value; TLC, total lung capacity.

### Cardiovascular biomarkers: Study group versus control group

The study group had a significantly lower RV-FAC and higher RV-GLS than the control group. Other 2D and speckle-tracking TTE measurements did not differ between the groups. Results are presented in Table 2.

**Table 2.** Comparison of RV functional measurements in 2D and speckle-tracking TTE between COPD patients and non-COPD controls.

Measurement	COPD (*n* = 47^†^)	Non-COPD (*n* = 23)	*p*-Value*
RV diameter^¶^, mm	36.7 ± 4.9	36.4 ± 4.5	0.772
RV-FAC, %	40.8 ± 5.4	48.6 ± 7.2	<0.001*
RV-S’, cm/s	14.0 (12.0–15.0)	14.0 (12.8–16.0)	0.688
RV-FWS^§^, %	24.6 (20.9–26.0)	26.1 (24.6–28.0)	0.018*
RV-GLS^§^, %	21.3 ± 2.5	22.2 ± 2.8	0.202

* *p*-Value < 0.05 indicates statistically significant results.

¶ Basal diameter, measured at end-diastole.

† RV-FAC, RV strains were obtained from 44 and 41 study group subjects, respectively.

§ The higher value indicating the better result (explained in Methods).

COPD, chronic obstructive pulmonary disease; FAC, fractional area change; FWS, free-wall strain; GLS, global longitudinal strain; RV, right ventricle; S’, tissue Doppler–derived tricuspid annular systolic velocity.

The median serum troponin-I concentration in patients with COPD (*n* = 47) was 1.12 pg/mL (IQR 0.99–1.56). Serum troponin-I levels were above the LOD in 44 (93.6%) patients with COPD and in 13 (56.5%) non-COPD subjects (*p* < 0.001; odds ratio 11.28, 95% confidence interval 2.70–47.19). Because a high proportion of control subjects had serum troponin-I levels below the LOD, we assumed that the median serum troponin-I concentration could not be reliably calculated for this group.

### Pulmonary function testing: From baseline to 12-week follow-up

In 34 patients with moderate-to-severe COPD who completed pulmonary function testing at baseline and at the 12-week follow-up, significant improvements were observed in several pulmonary function parameters. Specifically, spirometric measures, including FEV_1_ and FVC (absolute and % pred.), increased, while residual volume-to-TLC and FRC/TLC (absolute and % pred.), decreased (Table 3).

**Table 3.** Changes in spirometry and body plethysmography parameters from baseline to 12-week follow-up.

Measurement	Baseline (*n* = 34)	12 weeks (*n* = 34)	*p*-Value*
FEV_1_, % pred.	60.62 ± 13.37	68.26 ± 14.40	<0.001*
FEV_1_, L	2.07 ± 0.59	2.32 ± 0.66	<0.001*
FVC, % pred.	79.15 ± 14.02	82.44 ± 14.82	0.012*
FVC, L	3.48 ± 0.88	3.60 ± 0.87	<0.001*
Residual volume, % pred.	155.50 (137.75–177.75)	144.00 (121.25–167.50)	0.059
Residual volume, L	3.68 ± 0.98	3.49 ± 0.81	0.154
Residual volume-to-TLC, % pred.	132.50 (123.75–149.00)	123.00 (114.00–141.25)	0.002*
Residual volume-to-TLC, %	51.35 ± 7.71	48.17 ± 6.88	0.003*
FRC, % pred.	128.24 ± 33.47	122.47 ± 26.39	0.070
FRC, L	4.39 ± 1.15	4.21 ± 0.95	0.111
FRC/TLC, % pred.	119.76 ± 15.08	113.62 ± 14.87	0.008*
FRC/TLC, %	61.57 ± 8.25	58.55 ± 7.49	0.007*
TLC, % pred.	105.91 ± 19.29	106.94 ± 14.29	0.619
TLC, L	7.10 ± 1.44	7.19 ± 1.29	0.519

* *p*-Value < 0.05 indicates statistically significant results.

FEV_1_, forced expiratory volume in 1 second; FRC, functional residual capacity; FVC, forced vital capacity; % pred., percentage of predicted value; TLC, total lung capacity.

### Right ventricular function: From baseline to 12-week follow-up

A total of 34 patients with COPD underwent conventional 2D TTE at both study visits, of whom 29 also underwent 2D speckle-tracking TTE. Conventional RV parameters, including RV basal diameter, RV-FAC, and RV-S′, did not change significantly over time. In contrast, significant improvements were observed in RV-GLS and RV-FWS. In a subgroup analysis restricted to patients with moderate COPD, similar significant improvements were also observed in RV-GLS and RV-FWS, whereas no significant changes were detected in conventional parameters. The results are presented in Table 4.

**Table 4.** Changes in 2D and speckle-tracking TTE measurements from baseline to 12-week follow-up.

Measurement	Baseline	12 weeks	*p*-Value*
Moderate-to-severe (*n* = 34^†^)			
RV diameter^¶^, mm	36.0 (32.8–38.5)	36.0 (33.8–37.0)	0.674
RV-FAC, %	39.4 (36.8–44.5)	40.0 (37.6–44.5)	0.416
RV-S’, cm/s	14.3 ± 2.3	13.8 ± 2.0	0.204
RV-FWS^‡^, %	24.2 ± 3.3	26.1 ± 3.6	0.003*
RV-GLS^‡^, %	21.7 ± 2.6	23.0 ± 2.8	0.025*
Moderate (*n* = 30^‡^)			
RV diameter, mm	36.0 (32.8–38.5)	36.0 (33.8–37.0)	0.310
RV-FAC, %	39.7 (37.3–45.5)	40.0 (37.5–44.9)	0.513
RV-S’, cm/s	14.4 ± 2.3	13.9 ± 2.1	0.204
RV-FWS^§^, %	23.9 ± 3.3	26.0 ± 3.7	0.003*
RV-GLS^§^, %	21.4 ± 2.3	22.7 ± 2.7	0.032*

* *p*-Value < 0.05 indicates statistically significant results.

¶ Basal diameter, measured at end-diastole.

† RV-FAC was obtained from 28 patients, RV-FWS and RV-GLS were obtained from 29 patients.

‡ RV-FAC was obtained from 25 patients, RV-FWS and RV-GLS were obtained from 27 patients.

§ The higher value indicating the better result (explained in Methods).

FAC, fractional area change; FWS, free-wall strain; GLS, global longitudinal strain; RV, right ventricle; S’, tissue Doppler–derived tricuspid annular systolic velocity.

**Figure 2.**
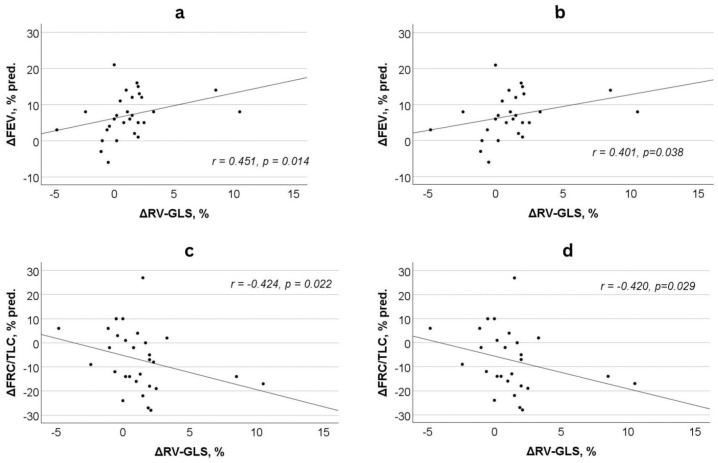
Correlations between the changes in RV-GLS measured by 2D speckle-tracking TTE and pulmonary function parameters ((a) ΔRV-GLS versus ΔFEV_1_ in patients with moderate-to-severe COPD, (b) ΔRV-GLS versus ΔFEV_1_ in patients with moderate COPD, (c) ΔRV-GLS versus ΔFRC/TLC in patients with moderate-to-severe COPD, (d) ΔRV-GLS versus ΔFRC/TLC in patients with moderate COPD). FEV_1_, forced expiratory volume in 1 second; FRC/TLC, functional residual capacity to total lung capacity ratio; % pred., percentage of predicted value; RV-GLS, right ventricular global longitudinal strain.

We found moderate positive correlations between changes in RV-GLS and FEV_1_ (absolute values and % pred.), as well as moderate negative correlations between changes in RV-GLS and FRC/TLC (absolute values and % pred.). Correlations were significant in both moderate-to-severe COPD and moderate COPD groups (Figure 2(a)–(d)).

We performed RV analysis using cardiac MRI in 29 patients at baseline and after 12 weeks of treatment. No significant changes were observed in RV-EDVi, RV-ESVi, RV-SVi, RV-EF, RV-FWS, or RV-GLS (all *p* > 0.05). However, a subgroup analysis of patients with moderate COPD (*n* = 25) revealed a significant change in RV-GLS, improving from 22.6 ± 6.1% to 25.5 ± 7.5% (*p* = 0.037). Meanwhile, no significant changes were detected in the remaining cardiac MRI parameters, including RV-FWS. Results are presented in Table 5.

**Table 5.** Changes in cardiac MRI measurements from baseline to 12-week follow-up.

Measurement	Baseline	12 weeks	*p*-Value*
Moderate-to-severe (*n* = 29)			
RV-EDVi, mL/m^2^	71.8 ± 19.4	72.6 ± 18.8	0.663
RV-ESVi, mL/m^2^	31.1 ± 13.0	30.6 ± 8.8	0.694
RV-SVi, mL/m^2^	43.0 (34.5–49.0)	38.0 (36.0–47.0)	0.633
RV-EF, %	58.0 ± 8.1	58.1 ± 5.7	0.941
RV-FWS^§^, %	29.3 ± 6.8	30.6 ± 7.7	0.337
RV-GLS^§^, %	22.9 (19.2–26.6)	24.0 (21.0–27.3)	0.214
Moderate (*n* = 25)			
RV-EDVi, mL/m^2^	71.8 ± 20.2	73.3 ± 19.8	0.458
RV-ESVi, mL/m^2^	30.8 ± 13.7	30.4 ± 9.2	0.828
RV-SVi, mL/m^2^	41.3 ± 9.3	42.8 ± 12.1	0.385
RV-EF, %	58.7 ± 8.4	58.6 ± 5.9	0.921
RV-FWS^§^, %	29.4 ± 7.0	31.5 ± 7.6	0.138
RV-GLS^§^, %	22.6 ± 6.1	25.5 ± 7.5	0.037*

* *p*-Value < 0.05 indicates statistically significant results.

§ The higher value indicating the better result (explained in Methods).

EDV, end-diastolic volume; EF, ejection fraction; ESV, end-systolic volume; FWS, free-wall strain; GLS, global longitudinal strain; i, indexed for body surface area; RV, right ventricle; SV, stroke volume.

### Serum troponin-I analysis: From baseline to 12-week follow-up

**Figure 3.**
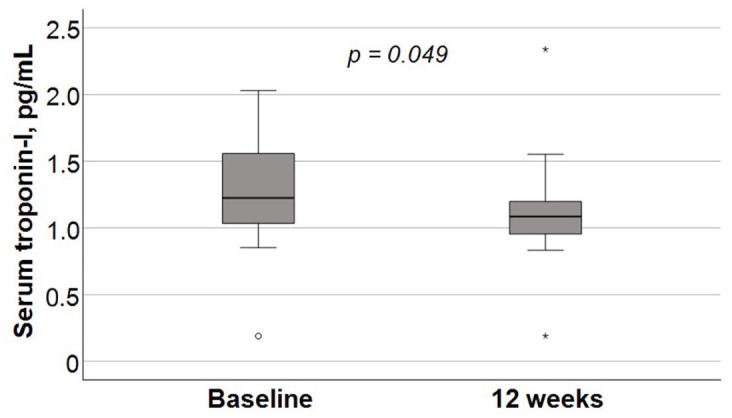
Changes in serum troponin-I levels in patients with moderate COPD from baseline to 12-week follow-up. Boxes represent the IQR with the median indicated by the horizontal line; whiskers extend to the most extreme values within 1.5 × IQR. Circle (○) represents outlier (1.5–3 × IQR), and asterisks (*) represent extreme outliers (>3 × IQR).

During the treatment period, median serum troponin-I levels in patients with moderate-to-severe COPD (*n* = 32) decreased from 1.17 pg/mL (IQR 1.00–1.52) to 1.09 pg/mL (IQR 0.95–1.21); however, this change did not reach statistical significance (*p* = 0.153). When a subgroup analysis of patients with moderate COPD (*n* = 28) was performed, serum troponin-I levels decreased from 1.23 pg/mL (IQR 1.03–1.56) to 1.09 pg/mL (IQR 0.95–1.20), reaching statistical significance (*p* = 0.049; Figure 3).

## Discussion

### Cardiovascular biomarkers: Comparison between study and control groups

While the mean RV-FAC and median RV-FWS values were within the normal range in both groups, patients with moderate-to-severe COPD had worse RV-FAC and RV-FWS in 2D and speckle-tracking TTE than the control group with comparable age, sex, BMI, and CVD profiles. According to literature, RV-FAC < 35% indicates RV systolic dysfunction, and RV-FWS is likely abnormal when >−20% (i.e., <20% in absolute numbers).^27^ The results of our study show a similar trend to previously published data. In the study by Nasir et al., measurements indicating RV dysfunction were compared between patients with COPD (*n* = 84) and healthy controls (*n* = 40). The authors found that the right index of myocardial performance (RIMP), TAPSE, RV-S′, RV-FAC, and RV basal strain were significantly worse in COPD patients. Similar to our study, both groups had mean FAC values above the lower limit of normal. Although RV-GLS and RV-FWS were not assessed, mean RV basal strain values exceeded 20% in both groups. In our study, TAPSE was not measured in the majority of COPD patients, precluding its inclusion in the analysis. We also speculate that the greater number of impaired parameters observed in the COPD group in their study may be due to the exclusion of patients with concomitant CVDs.^29^ In a recently published study by Hojda et al., patients with COPD (*n* = 55) demonstrated significantly better RV functional measurements than healthy controls (*n* = 15), including TAPSE, RV-S′, RV-FAC, RV-FWS, and RV 4-chamber longitudinal strain (4CLS). It is worth mentioning, however, that most mean values in the COPD group were below the lower limit of normal, particularly RV-FAC, RV-FWS, and RV-4CLS. These findings may be due to differences in study populations, as patients across GOLD stages 1 to 4 were included in their study.^23^ Similarly, in another study, patients with COPD (*n* = 32) had lower mean RV-FWS and RV-4CLS compared with controls (*n* = 37), whereas TAPSE did not differ between groups. RV-FAC and RV-S′ were not evaluated. RV-FWS and RV-4CLS values in the COPD group were markedly lower than those observed in our cohort (16.53 ± 5.89% and 14.65 ± 4.53%, respectively), which may be explained by differences in inclusion criteria, as patients across all GOLD stages were included in that study as well.^15^ While differences in RV functional parameters may have appeared more pronounced in other studies, it should be noted that our analysis compared COPD patients of GOLD stages 2–3 with CVDs to control subjects closely matched for age, sex, BMI, and CVD profiles. This may have influenced the magnitude of observed differences in our cohort.

When serum troponin-I levels were compared between COPD patients and controls in our study, a significantly higher proportion of COPD patients had values above the LOD (>1.38 pg/mL), despite similar age, sex, BMI, and CVD profiles. These findings are consistent with previous reports. For example, Weir-McCall et al. demonstrated significantly higher high-sensitivity troponin-I concentrations in individuals with COPD (*n* = 58) compared with healthy volunteers (*n* = 21) (2.27 ± 1.90 pg/mL vs 0.92 ± 0.49 pg/mL; *p* < 0.001). In contrast to our study, their analysis excluded participants with CVDs and included COPD patients across all GOLD stages.^30^ In a large population-based cohort, Nilsson et al. observed that both median high-sensitivity troponin-I concentrations and the proportion of individuals with levels above the predefined threshold (⩾5 ng/L) were higher in patients with COPD (*n* = 601) than in those with normal lung function (*n* = 755). Median high-sensitivity troponin-I concentrations were 3.3 ng/L (IQR 3.6) in the COPD group and 3.2 ng/L (IQR 2.9) in participants with normal lung function (*p* = 0.019). High-sensitivity troponin-I levels above the threshold were observed in 31.1% of COPD patients compared with 24.9% of individuals with normal lung function (*p* = 0.011). While the COPD group in this study also included patients across GOLD stages 1–4, individuals with CVDs were not excluded.^31^ It is also important to note that we used a contemporary serum troponin-I assay, which may explain why concentrations could not be measured accurately in more than half of the non-COPD subjects.

### Changes in lung function measurements with dual bronchodilation

The beneficial effects of LAMA/LABA therapy, including TIO/OLO, on lung function assessed by spirometry and body plethysmography are well established.^32–35^ Several aspects of the changes in lung function observed in our study require further discussion.

Firstly, FEV_1_ improved significantly by 250 ± 213 mL. The magnitude of this change may be explained by our measurement methodology: while FEV_1_ in clinical trials is typically assessed as trough FEV_1_ or as FEV_1_ area under the curve over 0–3 h (AUC_0-3_), we measured FEV_1_ 2 hours after morning inhalation, thereby capturing near-peak bronchodilator response. For example, in the OTEMTO 1 and 2 trials, which evaluated 12 weeks of TIO/OLO 5/5 μg in patients with moderate-to-severe COPD, FEV_1_ AUC_0-3_ improved markedly compared with placebo (331 mL and 299 mL in OTEMTO 1 and 2, respectively; *p* < 0.0001), whereas improvements in trough FEV_1_ were more modest (162 mL and 166 mL, respectively; *p* < 0.0001).^36^ Moreover, the magnitude of FEV_1_ improvement in our cohort may also be influenced by the inclusion of newly diagnosed, treatment-naïve patients, who may show a greater response to bronchodilator therapy.

Secondly, although the change in residual volume did not reach statistical significance during the study period, a trend toward reduction of % pred. value was observed (*p* = 0.059), which may partly reflect the limited sample size and statistical power. In addition, other parameters of air trapping and hyperinflation, such as residual volume-to-TLC ratio and FRC/TLC, improved significantly. This may also reflect the limited ability of absolute volume measurements alone to detect early improvement in lung mechanics, whereas relative parameters may better represent these changes.^37^ Together, our findings suggest that the treatment improved lung mechanics despite the lack of statistical significance in the change in residual volume.

### Changes in right ventricular function measurements with dual bronchodilation

There has recently been increasing interest in the potential impact of inhaled therapies for COPD on cardiac function. In our study, we evaluated the effects of TIO/OLO 5/5 µg FDC once daily prescribed as routine clinical care on RV function and found significant improvements in RV-FWS and RV-GLS after 12 weeks of treatment in patients with moderate-to-severe COPD, as well as in the subgroup of patients with moderate COPD, as assessed by 2D speckle-tracking TTE. However, no changes in conventional RV measurements were observed. In addition, RV-GLS improved in the moderate COPD subgroup when assessed by cardiac MRI. While the changes in RV strain were statistically significant, the improvements were relatively modest, and their clinical significance remains uncertain, particularly as baseline values were within the normal range.

Several studies have evaluated the effects of LAMA/LABA therapy on cardiac function; however, these investigations have predominantly focused on ventricular volumes. Herth et al. conducted an exploratory, randomized, double-blind, double-dummy, multicenter, crossover study in which cardiac functional parameters were assessed and compared in 76 patients using cardiac MRI after 6 weeks of treatment with TIO/OLO versus fluticasone propionate/salmeterol. Although an improvement in left ventricular (LV) EDVi was observed, this effect did not differ between treatment groups, and no significant changes were detected in RV functional measurements. Similar to our study, their analysis included RV-EDVi, RV-ESVi, RV-SVi, and RV-EF with the additional assessment of RV mass index.^11^ Hohlfeld et al. carried out a double-blind, randomized, two-period crossover, placebo-controlled, single-center study involving 62 participants, in which cardiac functional measurements were assessed using cardiac MRI after 14 days of treatment with indacaterol/glycopyrronium versus placebo. Although the primary endpoint was the change in LV-EDVi, RV measurements were also evaluated. In addition to improvements in LV-EDVi, treatment with indacaterol/glycopyrronium was associated with significant increases in RV-EDVi and RV-SVi compared with placebo. However, this study included only COPD patients with lung hyperinflation, defined as a baseline residual volume >135% pred., which may have influenced the detection of significant changes in cardiac volumes.^10^

To our knowledge, there are no published studies in which RV strain has been evaluated during COPD treatment with LAMA/LABA combinations (or other inhaled therapies). However, in a previously mentioned prospective, observational, comparative study by Nasir et al., RV basal strain was assessed at baseline (*n* = 84) and after 6 months of follow-up (*n* = 67) and was found to change significantly from −21.9 ± 5.45% to −21.6 ± 5.55% (*p* < 0.01). They also found that RV basal strain showed moderate negative correlations with FEV_1_ at baseline and after 6 months; it is important to note that, differently from our study, this inverse relationship reflects the expression of strain values as negative numbers. Conventional RV parameters, including RIMP, RV-FAC, and RV free-wall thickness, also changed significantly (all *p* < 0.01). Importantly, in contrast to the trends observed in our study, RV parameters deteriorated over the observation period. However, the study evaluated patients after 6 months without specifying the treatments prescribed or whether patients were adherent to their therapies.^29^ It is also worth mentioning the study by Kanar et al., in which 46 patients with COPD who underwent a 3-month pulmonary rehabilitation program demonstrated significant improvements in RV-FWS increasing from 18.1 ± 3.4% to 22.9 ± 3.7% (*p* < 0.001), and in RV-GLS improving from 20.4 ± 2.4% to 21.9 ± 2.9% (*p* < 0.001), as assessed by 2D speckle-tracking TTE. Among conventional echocardiographic measurements, only TAPSE and the tissue Doppler myocardial performance index showed significant improvements.^38^ Taken together, the findings of Kanar et al. and our study highlight the value of RV strain assessment in detecting treatment-related changes in RV function. While conventional RV functional parameters may remain stable, improvements in RV strain might reflect subclinical changes.^39^ However, clinically meaningful thresholds for RV strain improvement in COPD are not yet established.

We also found that improvement in RV-GLS measured by 2D speckle-tracking TTE correlated significantly with improvements in several pulmonary function parameters, particularly FEV_1_ and FRC/TLC. While data regarding the relationship between lung function changes and RV strain during dual bronchodilation are limited, an inverse correlation between change in residual volume and change in RV-EDVi (*r* = −0.38, *p* = 0.01) was reported in the study by Hohlfeld et al.^10^ From a clinical perspective, our findings support previous evidence that improvement in lung mechanics may lead to favorable changes in cardiac function. This relationship may be explained by reduced intrathoracic pressures, which can enhance RV loading and pulmonary vascular hemodynamics, resulting in improved RV strain.

### Changes in serum troponin-I concentration with dual bronchodilation

In our study, the change in serum troponin-I concentration after 12 weeks of treatment did not reach statistical significance in the overall group of patients with moderate-to-severe COPD. However, a modest but statistically significant decrease was observed in the subgroup of patients with moderate COPD (*p* = 0.049).

Higher troponin-I concentrations, even within the normal range, have been reported to be associated with RV dysfunction and increased all-cause mortality in patients with stable COPD.^24^ Nevertheless, studies evaluating the dynamics of troponin-I during treatment with inhaled therapies remain insufficient. We identified one double-blind randomized controlled trial by Adamson et al., in which plasma high-sensitivity troponin-I was measured in 1599 patients before and after 3 months of treatment with placebo, fluticasone furoate, vilanterol, or their combination. In that study, troponin-I levels were unaffected by inhaled therapies at follow-up (*p* > 0.05).^40^ Although direct comparison between our relatively small cohort and this large trial is limited, we hypothesize that the reduction in serum troponin-I observed in our study may be related to improved lung deflation following dual bronchodilation, potentially resulting in decreased RV afterload and myocardial wall stress, as well as improved oxygenation. However, this borderline finding should be interpreted with caution, given the exploratory nature of the analysis, the limited sample size, and the absence of adjustment for multiple comparisons.

## Strengths and limitations of our study

To our knowledge, this is the first study to evaluate changes in RV strain and serum troponin-I during stable COPD treatment with inhaled medications. Moreover, real-world studies evaluating the effects of initial COPD therapy in treatment-naïve patients are rarely performed. Such studies are important, as they allow evaluation of the true baseline disease state and provide a more accurate linkage of observed changes to the initiated therapy, without the influence of prior treatment.

This study has several important limitations. First, as a single-center real-world study with a relatively small sample size and no formal a priori power calculation, the analyses of the main outcomes, including both RV strains and serum troponin-I, should be considered exploratory and hypothesis-generating. Additionally, no adjustment for multiple comparisons was applied, which may increase the possibility of type I error. Furthermore, no adjustment for potential confounding was performed. Therefore, residual confounding cannot be excluded.

Second, a proportion of patients did not attend the 12-week follow-up visit, which is common in prospective real-world observational studies and reflects routine clinical practice. In addition, some patients were referred for cardiovascular evaluation after baseline assessment, which led to the initiation of new pharmacological treatment and precluded their further participation in the study.

Third, the study lacked a control group for longitudinal comparisons, which did not allow for assessing changes in outcomes across different COPD treatment options.

Finally, sample sizes varied across analyses due to incomplete imaging and laboratory data. In particular, 2D speckle-tracking TTE measurements were limited by suboptimal acoustic windows in COPD patients with lung hyperinflation, resulting in smaller sample sizes for RV-FAC and RV strain analyses and potentially introducing selection bias. Similar limitations applied to cardiac MRI and follow-up serum troponin-I measurements, which were not available in all participants due to patient-related, technical, and sample-related factors.

These issues may limit the generalizability of the findings and should be considered when interpreting the results.

## Conclusion

COPD significantly affects RV function and serum troponin-I levels, regardless of concomitant CVDs. Initiation of dual bronchodilation improves lung function and is associated with beneficial effects on RV strain and serum troponin-I in patients with newly diagnosed, treatment-naïve COPD. Improvements in RV strain during dual bronchodilation may be related to enhanced lung function. Prompt initiation of treatment might play an important role in achieving RV functional improvement. These findings should be interpreted cautiously because of the observational design and limited sample size, and require confirmation in further studies.

## Supplemental Material

sj-docx-3-tar-10.1177_17534666261452491 – Supplemental material for Effects of dual bronchodilation on right ventricular function and troponin-I in newly diagnosed, moderate-to-severe chronic obstructive pulmonary disease: a prospective real-world observational study Supplemental material, sj-docx-3-tar-10.1177_17534666261452491 for Effects of dual bronchodilation on right ventricular function and troponin-I in newly diagnosed, moderate-to-severe chronic obstructive pulmonary disease: a prospective real-world observational study by Ieva Dimiene, Gintare Neverauskaite-Piliponiene, Paulius Bucius, Paulius Simkus, Lina Padervinskiene, Airidas Rimkunas, Egle Ereminiene and Skaidrius Miliauskas in Therapeutic Advances in Respiratory Disease

sj-docx-4-tar-10.1177_17534666261452491 – Supplemental material for Effects of dual bronchodilation on right ventricular function and troponin-I in newly diagnosed, moderate-to-severe chronic obstructive pulmonary disease: a prospective real-world observational study Supplemental material, sj-docx-4-tar-10.1177_17534666261452491 for Effects of dual bronchodilation on right ventricular function and troponin-I in newly diagnosed, moderate-to-severe chronic obstructive pulmonary disease: a prospective real-world observational study by Ieva Dimiene, Gintare Neverauskaite-Piliponiene, Paulius Bucius, Paulius Simkus, Lina Padervinskiene, Airidas Rimkunas, Egle Ereminiene and Skaidrius Miliauskas in Therapeutic Advances in Respiratory Disease

sj-docx-5-tar-10.1177_17534666261452491 – Supplemental material for Effects of dual bronchodilation on right ventricular function and troponin-I in newly diagnosed, moderate-to-severe chronic obstructive pulmonary disease: a prospective real-world observational study Supplemental material, sj-docx-5-tar-10.1177_17534666261452491 for Effects of dual bronchodilation on right ventricular function and troponin-I in newly diagnosed, moderate-to-severe chronic obstructive pulmonary disease: a prospective real-world observational study by Ieva Dimiene, Gintare Neverauskaite-Piliponiene, Paulius Bucius, Paulius Simkus, Lina Padervinskiene, Airidas Rimkunas, Egle Ereminiene and Skaidrius Miliauskas in Therapeutic Advances in Respiratory Disease

sj-docx-6-tar-10.1177_17534666261452491 – Supplemental material for Effects of dual bronchodilation on right ventricular function and troponin-I in newly diagnosed, moderate-to-severe chronic obstructive pulmonary disease: a prospective real-world observational study Supplemental material, sj-docx-6-tar-10.1177_17534666261452491 for Effects of dual bronchodilation on right ventricular function and troponin-I in newly diagnosed, moderate-to-severe chronic obstructive pulmonary disease: a prospective real-world observational study by Ieva Dimiene, Gintare Neverauskaite-Piliponiene, Paulius Bucius, Paulius Simkus, Lina Padervinskiene, Airidas Rimkunas, Egle Ereminiene and Skaidrius Miliauskas in Therapeutic Advances in Respiratory Disease

sj-docx-7-tar-10.1177_17534666261452491 – Supplemental material for Effects of dual bronchodilation on right ventricular function and troponin-I in newly diagnosed, moderate-to-severe chronic obstructive pulmonary disease: a prospective real-world observational study Supplemental material, sj-docx-7-tar-10.1177_17534666261452491 for Effects of dual bronchodilation on right ventricular function and troponin-I in newly diagnosed, moderate-to-severe chronic obstructive pulmonary disease: a prospective real-world observational study by Ieva Dimiene, Gintare Neverauskaite-Piliponiene, Paulius Bucius, Paulius Simkus, Lina Padervinskiene, Airidas Rimkunas, Egle Ereminiene and Skaidrius Miliauskas in Therapeutic Advances in Respiratory Disease

sj-jpg-1-tar-10.1177_17534666261452491 – Supplemental material for Effects of dual bronchodilation on right ventricular function and troponin-I in newly diagnosed, moderate-to-severe chronic obstructive pulmonary disease: a prospective real-world observational study Supplemental material, sj-jpg-1-tar-10.1177_17534666261452491 for Effects of dual bronchodilation on right ventricular function and troponin-I in newly diagnosed, moderate-to-severe chronic obstructive pulmonary disease: a prospective real-world observational study by Ieva Dimiene, Gintare Neverauskaite-Piliponiene, Paulius Bucius, Paulius Simkus, Lina Padervinskiene, Airidas Rimkunas, Egle Ereminiene and Skaidrius Miliauskas in Therapeutic Advances in Respiratory Disease

sj-jpg-2-tar-10.1177_17534666261452491 – Supplemental material for Effects of dual bronchodilation on right ventricular function and troponin-I in newly diagnosed, moderate-to-severe chronic obstructive pulmonary disease: a prospective real-world observational study Supplemental material, sj-jpg-2-tar-10.1177_17534666261452491 for Effects of dual bronchodilation on right ventricular function and troponin-I in newly diagnosed, moderate-to-severe chronic obstructive pulmonary disease: a prospective real-world observational study by Ieva Dimiene, Gintare Neverauskaite-Piliponiene, Paulius Bucius, Paulius Simkus, Lina Padervinskiene, Airidas Rimkunas, Egle Ereminiene and Skaidrius Miliauskas in Therapeutic Advances in Respiratory Disease

## Declarations

**Ethics approval and consent to participate:** This study was approved by the Kaunas Regional Biomedical Research Ethics Committee (No. BE-2-46, 14 April 2021, Kaunas, Lithuania). It was registered on ClinicalTrials.gov (ID NCT06072690). All participants provided written informed consent to participate in this study.

**Consent for publication:** All participants provided written informed consent prior to participation in this study, including consent for the publication of non-identifiable data.

**Availability of data and materials:** The data that support the findings of this study are available from the corresponding author upon reasonable request.Primary data from this study were previously presented as an abstract at the European Respiratory Society Congress 2025, held in Amsterdam, the Netherlands.
