# Supplementary material for: Effects of dual bronchodilation on right ventricular function and troponin-I in newly diagnosed, moderate-to-severe chronic obstructive pulmonary disease: a prospective real-world observational study
Source: Ther Adv Respir Dis. 2026 Jun 24;20:17534666261452491. doi: 10.1177/17534666261452491 (PMC13305909; doi:10.1177/17534666261452491)
Supplement: sj-docx-4-tar-10.1177_17534666261452491 – Supplemental material for Effects of dual bronchodilation on right ventricular function and troponin-I in newly diagnosed, moderate-to-severe chronic obstructive pulmonary disease: a prospective real-world observational study [file sj-docx-4-tar-10.1177_17534666261452491.docx]

**Supplementary Figure 2.** Assessment of the RV strain indices in MRI with feature tracking. More negative strain values indicate better results. In this study we used absolute values to ease statistical analysis (the higher number, the better result). Detailed methodology desribed in Methods (section titled “Cardiac magnetic resonance imaging with feature tracking”).
