# Supplementary material for: Effects of dual bronchodilation on right ventricular function and troponin-I in newly diagnosed, moderate-to-severe chronic obstructive pulmonary disease: a prospective real-world observational study
Source: Ther Adv Respir Dis. 2026 Jun 24;20:17534666261452491. doi: 10.1177/17534666261452491 (PMC13305909; doi:10.1177/17534666261452491)
Supplement: sj-docx-5-tar-10.1177_17534666261452491 – Supplemental material for Effects of dual bronchodilation on right ventricular function and troponin-I in newly diagnosed, moderate-to-severe chronic obstructive pulmonary disease: a prospective real-world observational study [file sj-docx-5-tar-10.1177_17534666261452491.docx]

**Supplementary Table 1.** Baseline characteristics of COPD patients who attended the 12-week follow-up visit (completers) versus those who did not (non-completers).

| **Characteristics** | **Completers (n=34)** | **Non-completers (n=13)** | ***p*-Value** |
| --- | --- | --- | --- |
| Age, years | 59.9 ± 8.2 | 64.5 ± 6.5 | 0.075 |
| Smoking exposure,  pack-years | 37.50 (18.75-50.00) | 27.00 (12.25-35.00) | 0.120 |
| BMI, kg/m^2^ | 27.3 ± 5.2 | 27.3 ± 3.5 | 0.979 |
| FEV_1_, % pred. | 60.62 ± 13.37 | 54.00 ± 13.44 | 0.137 |
| Eos ≥ 300 cells/µL, *n* (%) | 12 (35.3) | 4 (30.8) | 1.000 |
| Residual volume,  % pred. | 155.50 (137.75 – 177.75) | 135.00 (127.00 – 165.50) | 0.207 |
| Arterial hypertension, *n* (%) | 23 (67.6) | 8 (61.5) | 0.739 |
| Stable ischemic cardiac disease, *n* (%) | 5 (14.7) | 1 (7.7) | 1.000 |
| Dyslipidemia, *n* (%) | 16 (47.1) | 6 (46.2) | 0.956 |
| Arrhythmias in the past, *n* (%) | 2 (5.9) | 2 (15.4) | 0.304 |

Abbreviations: BMI – body mass index, FEV1 – forced expiratory volume in 1 second, % pred. – percentage of predicted value, Eos – blood eosinophil count
