# Supplementary material for: Effects of dual bronchodilation on right ventricular function and troponin-I in newly diagnosed, moderate-to-severe chronic obstructive pulmonary disease: a prospective real-world observational study
Source: Ther Adv Respir Dis. 2026 Jun 24;20:17534666261452491. doi: 10.1177/17534666261452491 (PMC13305909; doi:10.1177/17534666261452491)
Supplement: sj-docx-7-tar-10.1177_17534666261452491 – Supplemental material for Effects of dual bronchodilation on right ventricular function and troponin-I in newly diagnosed, moderate-to-severe chronic obstructive pulmonary disease: a prospective real-world observational study [file sj-docx-7-tar-10.1177_17534666261452491.docx]

**Supplementary Table 2.** STROBE Statement—Checklist of items that should be included in reports of cohort studies.

|  | Item No | Recommendation | Page  No | | Comment |
| --- | --- | --- | --- | --- | --- |
| **Title and abstract** | 1 | (*a*) Indicate the study’s design with a commonly used term in the title or the abstract | 1 | | “Prospective real-world observational study” is stated in the abstract. |
|  |  | (*b*) Provide in the abstract an informative and balanced summary of what was done and what was found | 1 | | Abstract clearly summarizes methods and key findings. |
| Introduction | | |  | |  |
| Background/rationale | 2 | Explain the scientific background and rationale for the investigation being reported | 2 | | The scientific background regarding the impact of COPD on the cardiovascular system, including right ventricular (RV) function and serum troponin I, is explained, along with previous findings on the effects of COPD treatments on cardiac function. |
| Objectives | 3 | State specific objectives, including any prespecified hypotheses | 2 | | Objectives are clearly stated. |
| Methods | | |  | |  |
| Study design | 4 | Present key elements of study design early in the paper | 2-4 | | Describes prospective cohort of COPD patients and controls. |
| Setting | 5 | Describe the setting, locations, and relevant dates, including periods of recruitment, exposure, follow-up, and data collection | 2-3 | | Newly diagnosed COPD cases confirmed at the Hospital of Lithuanian University of Health Sciences Kauno Klinikos in the Department of Pulmonology; recruitment period from September 2021 to October 2025; duration of the study 12 weeks. |
| Participants | 6 | (*a*) Give the eligibility criteria, and the sources and methods of selection of participants. Describe methods of follow-up | 3 | | COPD confirmed based on post-bronchodilator spirometry demonstrating a forced expiratory volume in FEV_1_/FVC ratio <0.7. Inclusion and exclusion criteria of COPD patients described in previously published article [25]; inclusion criteria of controls described in “Study design and participants” section |
|  |  | (*b*) For matched studies, give matching criteria and number of exposed and unexposed | N/A | | Not applicable, as this was an unmatched study; baseline characteristics of COPD patients and non-COPD controls were compared. |
| Variables | 7 | Clearly define all outcomes, exposures, predictors, potential confounders, and effect modifiers. Give diagnostic criteria, if applicable | 3-4 | | Outcomes (RV global longitudinal strain (GLS), free-wall strain (FWS), and serum troponin I) are defined. No additional exposures, predictors or confounders were included. COPD diagnosis was based on post-bronchodilator FEV₁/FVC < 0.7. |
| Data sources/ measurement | 8* | For each variable of interest, give sources of data and details of methods of assessment (measurement). Describe comparability of assessment methods if there is more than one group | 2-5 | | All variables were evaluated using standardized measurement protocols and consistent assessment methods. The same protocols and assessment methods were applied in the control group. |
| Bias | 9 | Describe any efforts to address potential sources of bias | 3, 13-14 | | Standardized measurement protocols were applied across groups and time points to minimize measurement bias. No adjustment for confounding was performed, and potential selection bias due to incomplete data and study design is acknowledged as a limitation. |
| Study size | 10 | Explain how the study size was arrived at | 3, 13 | | No formal a priori sample size calculation was performed. All eligible treatment-naïve COPD patients who consent to participate were included. |
| Quantitative variables | 11 | Explain how quantitative variables were handled in the analyses. If applicable, describe which groupings were chosen and why | 5 | | Quantitative variables were analysed as continuous variables and are presented as mean ± SD or median (IQR), as appropriate. Troponin I was categorized as below or above the limit of detection (LOD; 0.38 pg/mL) when comparing COPD and non-COPD groups. |
| Statistical methods | 12 | (*a*) Describe all statistical methods, including those used to control for confounding | 5 | | Detailed description of statistical methods is present. No adjustment for confounding was used. |
|  |  | (*b*) Describe any methods used to examine subgroups and interactions | 7, 9, 11 | | Exploratory subgroup analysis was performed in patients with moderate COPD, using the same statistical methods as described above. |
|  |  | (*c*) Explain how missing data were addressed | 5 | | Analyses were performed using available data for each variable; no imputation of missing values was performed. |
|  |  | (*d*) If applicable, explain how loss to follow-up was addressed | 3, Supplementary Table 1 | | Loss to follow-up was addressed by reporting the number of participants completing follow-up and comparing baseline characteristics between completers and non-completers to assess potential attrition bias. |
|  |  | (*e*) Describe any sensitivity analyses | N/A | | N/A |
| Results | | |  | |  |
| Participants | 13* | (a) Report numbers of individuals at each stage of study—eg numbers potentially eligible, examined for eligibility, confirmed eligible, included in the study, completing follow-up, and analysed | 3 | | 47 COPD patients and 23 controls were included; 34 COPD patients completed the 12-week follow-up. The detailed description is in the ‘Methods’ section. |
|  |  | (b) Give reasons for non-participation at each stage | 3 | | Reasons for non-participation in the 12-week follow-up are described in the ‘Methods’ section. |
|  |  | (c) Consider use of a flow diagram | 3 | | A flow diagram of participant selection and follow-up. |
| Descriptive data | 14* | (a) Give characteristics of study participants (eg demographic, clinical, social) and information on exposures and potential confounders | 6, Table 1 | | Baseline characteristics of COPD patients and controls are presented in Table 1, including demographic and variables. |
|  |  | (b) Indicate number of participants with missing data for each variable of interest | 4-5,  Table 2,  Table 4 | | Missing data for each variable of interest described in the ‘Methods’ section and under tables in the main text. |
|  |  | (c) Summarise follow-up time (eg, average and total amount) | 2 | | Follow-up duration (12 weeks) is described. |
| Outcome data | 15* | Report numbers of outcome events or summary measures over time | 5-7 | | Outcome data, including pulmonary function parameters, RV function, and serum troponin I, are reported at baseline and after 12 weeks. |
| Main results | 16 | (*a*) Give unadjusted estimates and, if applicable, confounder-adjusted estimates and their precision (eg, 95% confidence interval). Make clear which confounders were adjusted for and why they were included | 5-7,  tables 1-5,  Figures 2 (a-d), 3 | | Unadjusted estimates (means ± SD, medians (IQR), and *p*-values) are reported. No confounder-adjusted analyses were performed in this study. |
|  |  | (*b*) Report category boundaries when continuous variables were categorized | 5 | | Serum troponin I was categorized as below or above the LOD (0.38 pg/mL); other continuous variables were analysed without categorization. |
|  |  | (*c*) If relevant, consider translating estimates of relative risk into absolute risk for a meaningful time period | N/A | | N/A |
| Other analyses | 17 | Report other analyses done—eg analyses of subgroups and interactions, and sensitivity analyses | 7,  Tables 4 and 5,  Figure 2 (b,d),  Figure 3 | | Subgroup analyses of RV function measurements and serum troponin-I in moderate COPD patients were reported. |
| Discussion | | |  | |  |
| Key results | 18 | Summarise key results with reference to study objectives | 8-14 | | Key results, including lung function, RV function and troponin I findings, are summarized in relation to the study objectives. |
| Limitations | 19 | Discuss limitations of the study, taking into account sources of potential bias or imprecision. Discuss both direction and magnitude of any potential bias | 13 | | Detailed discussion about limitations of the study is present in the section ‘Strengths and limitations of our study’. |
| Interpretation | 20 | Give a cautious overall interpretation of results considering objectives, limitations, multiplicity of analyses, results from similar studies, and other relevant evidence | 8-14 | Cautious interpretation of results, including limitations and comparison with previous studies, is provided in the ‘Discussion’ section | |
| Generalisability | 21 | Discuss the generalisability (external validity) of the study results | 13-14 | | Generalisability of the findings may be limited by the small sample size, incomplete data, as well as by the real-world, single-centre cohort and exploratory design. |
| Other information | | |  | |  |
| Funding | 22 | Give the source of funding and the role of the funders for the present study and, if applicable, for the original study on which the present article is based | 14 | | This research was funded by the Science Foundation of the Lithuanian University of Health Sciences; no grant number was assigned. |

*Give information separately for exposed and unexposed groups.

**Note:** An Explanation and Elaboration article discusses each checklist item and gives methodological background and published examples of transparent reporting. The STROBE checklist is best used in conjunction with this article (freely available on the Web sites of PLoS Medicine at http://www.plosmedicine.org/, Annals of Internal Medicine at http://www.annals.org/, and Epidemiology at http://www.epidem.com/). Information on the STROBE Initiative is available at http://www.strobe-statement.org.
