# Supplementary figures and images for: Effects of dual bronchodilation on right ventricular function and troponin-I in newly diagnosed, moderate-to-severe chronic obstructive pulmonary disease: a prospective real-world observational study
Source: Ther Adv Respir Dis. 2026 Jun 24;20:17534666261452491. doi: 10.1177/17534666261452491 (PMC13305909; doi:10.1177/17534666261452491)

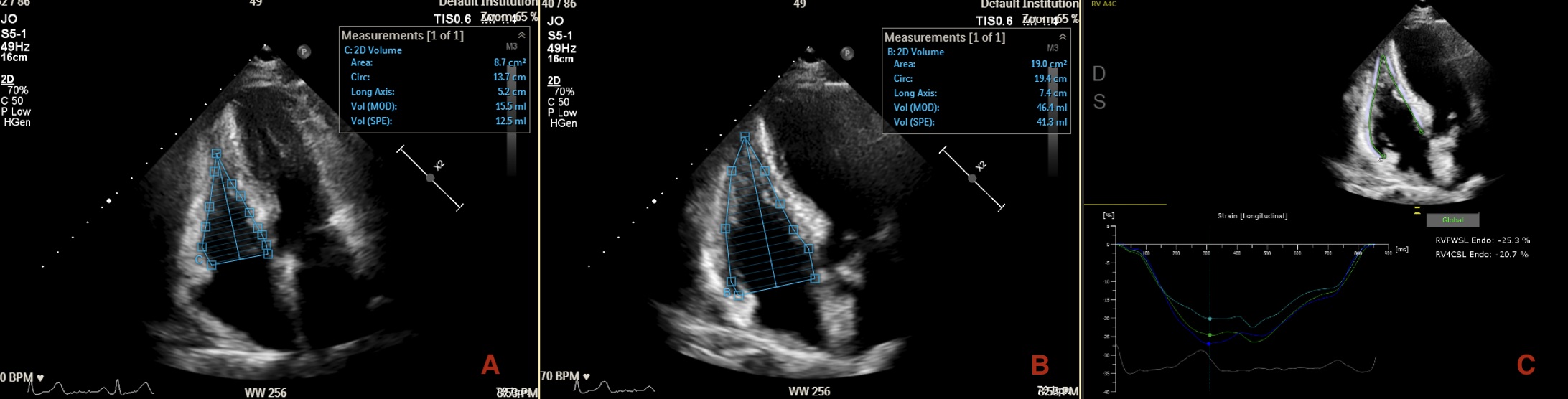

Supplement: sj-jpg-1-tar-10.1177_17534666261452491 – Supplemental material for Effects of dual bronchodilation on right ventricular function and troponin-I in newly diagnosed, moderate-to-severe chronic obstructive pulmonary disease: a prospective real-world observational study [file sj-jpg-1-tar-10.1177_17534666261452491.jpg]

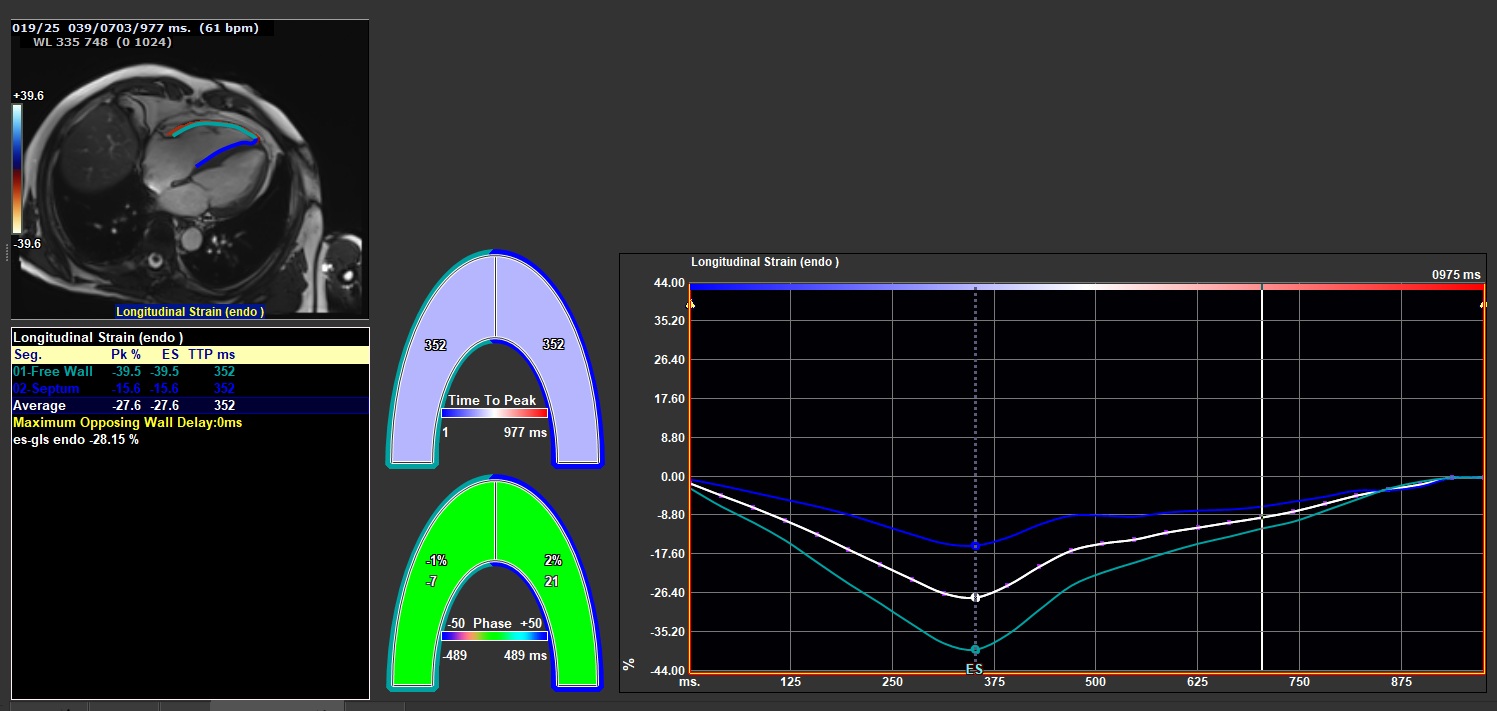

Supplement: sj-jpg-2-tar-10.1177_17534666261452491 – Supplemental material for Effects of dual bronchodilation on right ventricular function and troponin-I in newly diagnosed, moderate-to-severe chronic obstructive pulmonary disease: a prospective real-world observational study [file sj-jpg-2-tar-10.1177_17534666261452491.jpg]
